# Book Reviews

**Published:** 1872-12-01

**Authors:** 


### Book Reviews.

A Practical Treatise on Urinary and Renal Diseases, including Urinary Deposits. Illustrated by numerous cases and engravings. By William Roberts, M. D. Second American, from the revised and enlarged London edition. Philadelphia: Henry C. Lea, 1872. Jansen, McClurg & Co., Chicago.

This is a volume of 612 pages, constituting a very full and complete treatise on the subject of Urinary and Renal Diseases. The work is divided into three parts — part first, which is in a measure introductory to the other two, being devoted to the consideration of the physical and chemical properties of the urine, and the various alterations which it undergoes under different circumstances of health and disease. The methods of examining the urine for clinical purposes are also explained; and the appearances of urinary deposits, both as observed by the naked eye and under microscopical examination, are fully described and figured.

The second part treats of the "urinary diseases," viz.: diabetes insipidus, diabetes mellitus, gravel and calculus, and chylous urine.

The organic diseases of the kidneys form the subject of the third and largest part of the work.

The first English edition of this work as well as a separate American reprint, has been out of print for nearly three years past. This new and enlarged edition is certain therefore to meet with the favorable reception to which its merits justly entitle it.

The Chicago Medical Register and Directory, 1872–3, containing a description of the Medical Colleges, Hospitals, Infirmaries, Asylums, and Charitable Institutions, together with the Medical and other Scientific Associations of the entire State of Illinois. Edited and published by T. Davis Fitch, M. D., and Norman Bridge, M. D.; revised by the Presidents of the regular Medical Colleges and Societies of Chicago, and of the Illinois State Medical Society.

The publication of this work has been undertaken by the editors in the belief that such a book was both needed and desired by the medical profession of the State, and particularly of Chicago. The table of contents is as follows: American Medical Association, its origin and history; Illinois State Medical Society; City, County, and District Medical Societies; Medical Colleges, Hospitals, and Dispensaries of the City; Charitable Institutions, Societies, etc., of the City and State; Directory, including lists of the physicians in most of the principal cities and towns of the State, as well as a full list of regular physicians in the city of Chicago. A list of the regularly graduated dentists and pharmaceutists of Chicago is also given.

The editors have done their work well and faithfully, and are entitled to much credit for their endeavors to present to the profession a complete and reliable medical register. They have succeeded in condensing into a small compass a large amount of matter of value and importance to the physicians of the State. It is their intention to publish the Register annually, but the present volume is issued so near the beginning of 1873, that volume two will not be published before May, '74.

The Register and Directory can be obtained by addressing: "Medical Register, 296 West Monroe Street, Chicago," mailed free of postage on receipt of $2.00.

